# Bruton’s tyrosine kinase regulates gut immune homeostasis through attenuating Th1 response

**DOI:** 10.1038/s41419-021-03702-y

**Published:** 2021-04-30

**Authors:** Di Guan, Zixi Wang, Jianxin Huo, Shengli Xu, Kong-Peng Lam

**Affiliations:** 1 NUS Graduate School for Integrative Sciences & Engineering (NGS), National University of Singapore, Singapore, Singapore; 2 Bioprocessing Technology Institute, A*STAR (Agency for Science, Technology and Research), Singapore, Singapore; 3 Department of Microbiology and Immunology, Yong Loo Lin School of Medicine, National University of Singapore, Singapore, Singapore; 4 Singapore Immunology Network (SIgN), A*STAR (Agency for Science, Technology and Research), Singapore, Singapore; 5 Department of Physiology, Yong Loo Lin School of Medicine, National University of Singapore, Singapore, Singapore

**Keywords:** Inflammatory bowel disease, Inflammation, Mucosal immunology, Inflammation

## Abstract

Inflammatory bowel disease (IBD) is driven by multiple genetic and environmental risk factors. Patients with mutations in Bruton’s tyrosine kinase (BTK) is known to manifest high prevalence of intestinal disorders including IBD. Although BTK mediates the signaling of various immune receptors, little is known how BTK maintains the homeostasis of the gut immune system. Here, we show that BTK-deficiency promotes IBD progression in a mouse model of colitis. Interestingly, the increased colitis susceptibility of BTK-deficient mice is not caused by gut microbiota changes but rather arises from enhanced pro-inflammatory Th1 response. More importantly, we find the heightened Th1 response in BTK-deficient mice to result from both T cell-extrinsic and -intrinsic mechanisms. BTK-deficient dendritic cells secret elevated levels of the Th1-polarizing cytokine IL-12 and BTK-deficient T cells are inherently more prone to Th1 differentiation. Thus, BTK plays critical roles in maintaining gut immune homeostasis and preventing inflammation via regulating T-cell polarization.

## Introduction

Disorders in the gut immune system can trigger the development of inflammatory bowel disease (IBD) leading to hyper-active immune responses against gut commensals^1^. Human IBD is highly heterogeneous and driven by an interplay of genetic and environmental risk factors. Previous genome-wide association studies have revealed genetic variants harboring risk for IBD, the majority of which individually confers modest risk and with IBD development requiring the accumulation of multiple variants^1–4^. Indeed, there are limited examples of single variant causing IBD such as that of IL-10 receptor mutations^5^.

Increasing evidence suggests that certain genetic deficiency may alter gut immune mechanisms and transform gut microbiota from normal into colitogenic, which further exacerbates inflammatory responses. Investigations of IBD patients have revealed significant changes in their microbiota compared with healthy controls^1^. A study showed that the spontaneous colitis in *Rag2*^−/−^*Tbx21*^*−/−*^ mice was transmissible to *Rag2*^−/−^*Tbx21*^*+/+*^ and immunocompetent mice when their colitogenic gut microbiota was transferred^6^. On the other hand, there are genetic variants such as the *Nlrp3*^R258W^ inflammasome that protects against IBD development by inducing a homeostatic gut microbiota^7^.

A recent report showed that intestinal manifestations and IBD are prevalent in X-linked agammaglobulinemia (XLA) patients^8^ with loss-of-function mutations in *Btk* gene^9^, suggesting that *Btk-*deficiency is a genetic risk factor for IBD. The *Btk* gene encodes Bruton’s tyrosine kinase (BTK), a non-receptor protein tyrosine kinase present in the cytoplasm of various immune cell types^10,11^. BTK is important for B-cell development and activation by mediating pre-B and B cell-receptor signaling^9,12^. BTK also transduces signaling downstream of chemokine and Toll-like receptors (TLRs) in B and innate immune cells^13–15^. As BTK is critically involved in multiple signaling pathways in various immune cells, its deficiency is likely to cause gut immune disorders through a combination of multiple mechanisms. However, little has been explored in this regard. In particular, it remains to be confirmed if BTK-deficiency could lead to IBD development and if so, what mechanisms or pathways are involved.

In this study, we demonstrated that BTK-deficiency exacerbated DSS-induced colitis in the mouse. The propensity for colitis development in BTK-deficient mice is not due to shifts in their gut microbiota. We found that BTK-deficient mice had enhanced pro-inflammatory Th1 response in the gut, arising from both T cell-extrinsic and -intrinsic mechanisms. Our study provides new understanding of how BTK maintains gut immune homeostasis via regulating T cell polarization.

## Results

### BTK-deficient mice are more susceptible to DSS-induced colitis

**Fig. 1:**
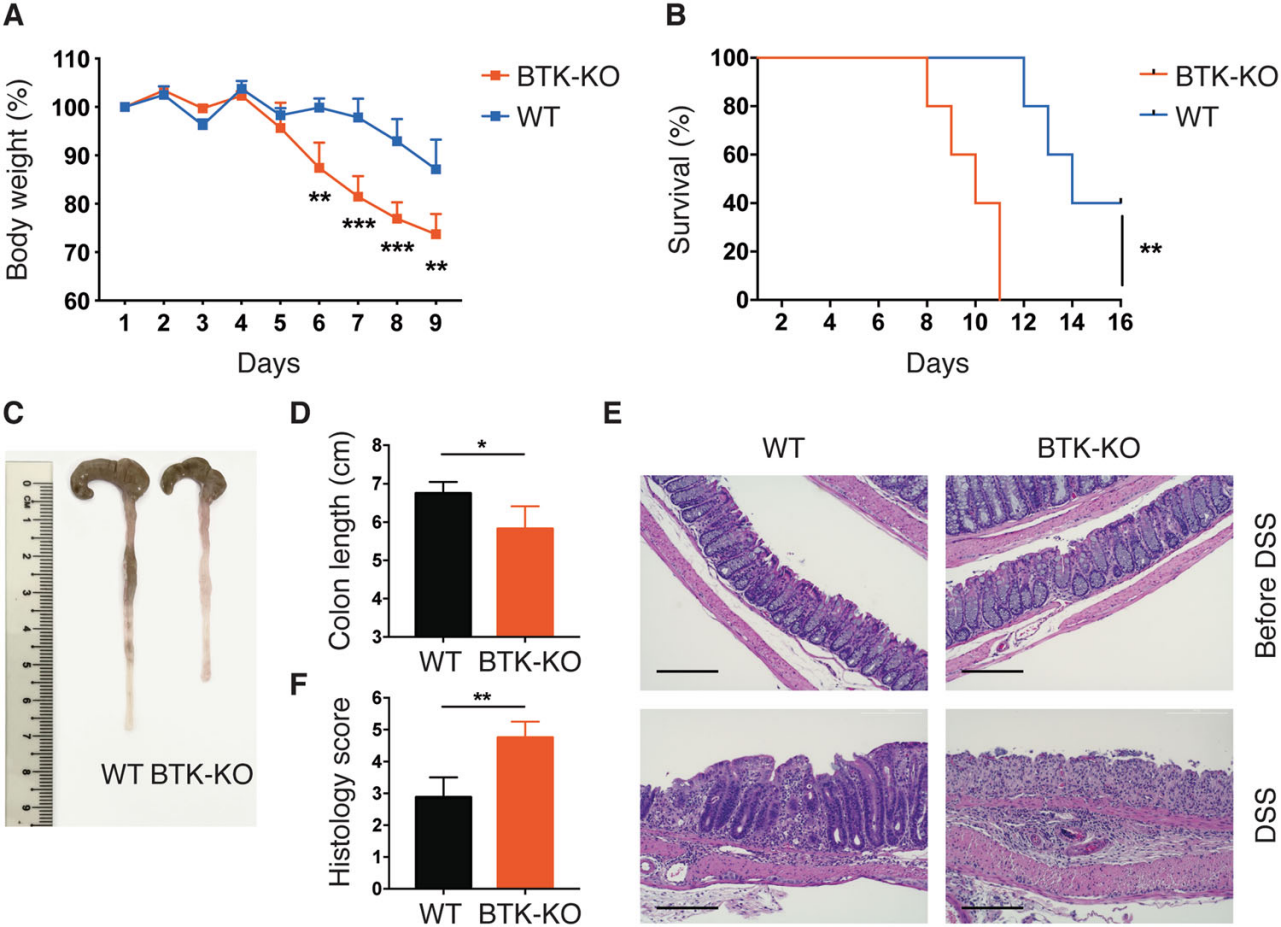
BTK-KO mice are more susceptible to DSS-induced colitis. Experimental colitis was induced in WT and BTK-KO mice by feeding mice ad libitum with drinking water containing 2% (w/v) DSS for 7 days. DSS-containing water was replaced by sterile drinking water from day 8 onwards. Body weight (**A**) and survival rate (**B**) of WT and BTK-KO mice were monitored every 24 h. **C**, **D** Colon lengths were measured upon euthanasia of DSS-treated mice on day 9. **E** Representative images of the H&E-stained colon sections from unchallenged and DSS-treated WT and BTK-KO mice; the scale bar is 200 μm. **F** The histology disease score of H&E-stained colon sections from DSS-treated WT and BTK-KO mice. Data are presented as mean ± SD (*n* = 5). Statistical significance in **A**, **D**, **F** was determined by two-sided independent sample Student’s *t*-test. Survival rate difference in **B** was determined by Log-rank (Mantel–Cox) test. *P < 0.05, **P < 0.01 and ***P < 0.001. Data shown are representative of two independent experiments.

To investigate the effect of BTK-deficiency on gut immune homeostasis, we subjected wild-type (WT) and *Btk* gene knock-out (BTK-KO) mice to DSS-induced acute colitis. As compared with WT controls, BTK-KO mice manifested early-onset colitis with significantly more weight loss (Fig. 1A), higher mortality rate (Fig. 1B) and greater shortening of colons (Fig. 1C, D). Histological analysis of BTK-KO colons also revealed more severe immune cell infiltration and intestinal architecture damage when compared with WT samples (Fig. 1E, F). Collectively, these data indicate that BTK-KO mice developed more severe colitis than WT mice when treated with DSS.

**Fig. 2:**
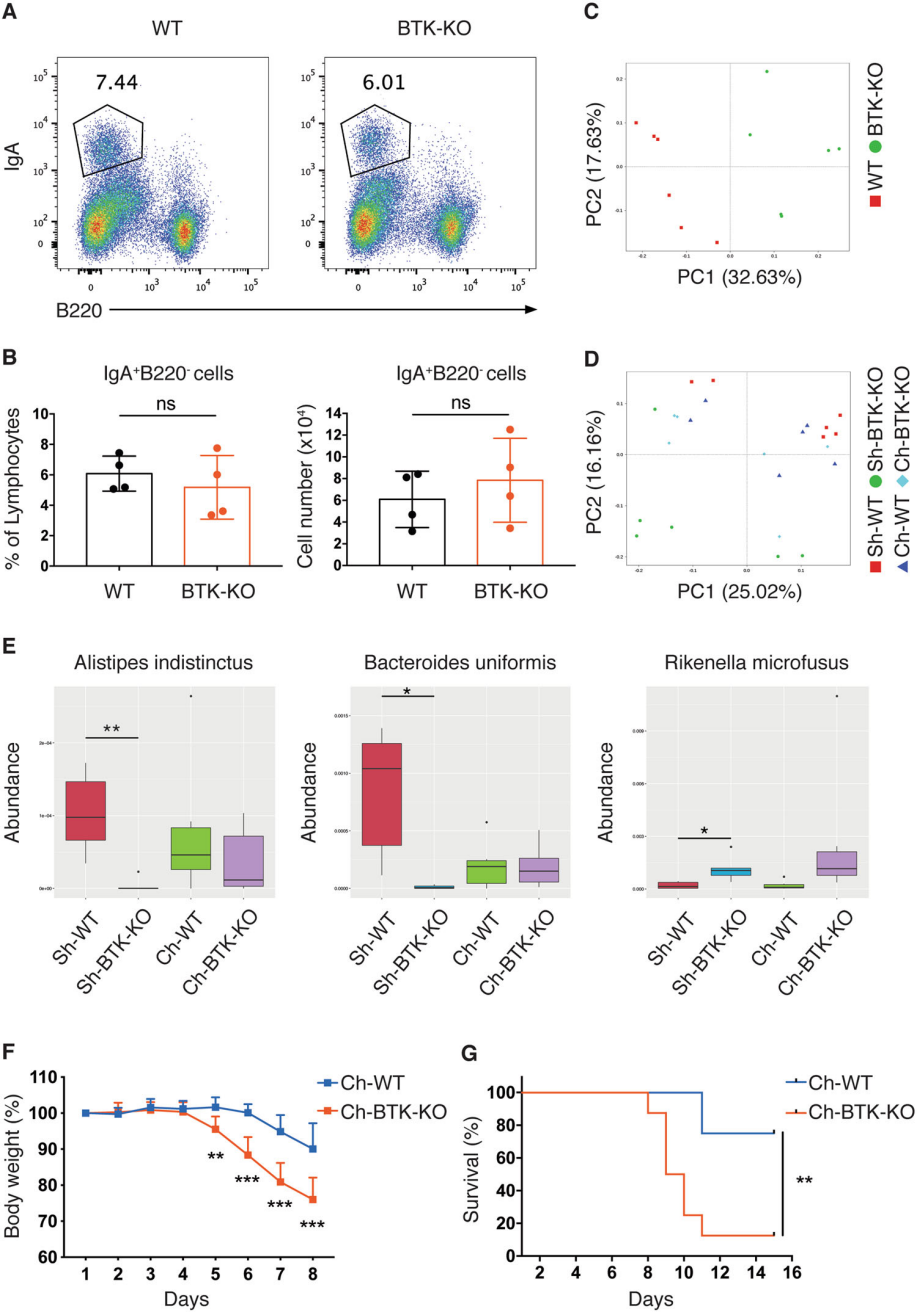
Increased colitis severity in BTK-KO mice is not due to gut microbiota shift. **A** Colon LP lymphocytes from WT and BTK-KO mice stained as indicated to reveal IgA-expressing B cells (IgA^+^B220^-^). Numbers shown are percentage of cells in the indicated gates. **B** Frequency (left) and total number (right) of IgA-expressing B cells in lymphocytes from colon LP of WT and BTK-KO mice. **C**–**E** Fecal bacteria composition of WT and BTK-KO mice analyzed by 16 S rRNA gene V3–V4 region sequencing. PCoA plot (based on unweighted UniFrac distance) of fecal bacteria was generated for singly-housed WT and singly-housed BTK-KO mice (**C**), and for singly-housed WT (Sh-WT), singly-housed BTK-KO (Sh-BTK-KO), co-housed WT (Ch-WT) and co-housed BTK-KO (Ch-BTK-KO) mice (**D**). **E** Metastat analysis of fecal bacteria species differentially abundant between WT and BTK-KO mice before and after cohousing. **F**, **G** WT and BTK-KO mice were cohoused at 1:1 ratio for 4 weeks. Experimental colitis was induced in co-housed BTK-KO (Ch-BTK-KO) and co-housed WT (Ch-WT) mice by giving drinking water containing 2% (w/v) DSS for 7 days. Body weight (**F**) and survival rate (**G**) of Ch-WT and Ch-BTK-KO mice were monitored every 24 h. Data are presented as mean ± SD for **B** and **F**, median with interquartile range for **E** [*n* = 4 for **B**, 6 for **C**–**E** and 8 for **F**, **G**]. Statistical significance in **B**, **F** was determined by two-sided independent sample Student’s *t*-test. Survival rate difference in **G** was determined by Log-rank (Mantel–Cox) test. ns: not significant; *P < 0.05, **P < 0.01 and ***P < 0.001 for **B**, **F**, **G**; *q < 0.05, **q < 0.01 for **E**.

Recent studies demonstrated that gut microbiota dysbiosis is a driving factor of gut inflammation^1,16,17^. Hence, we assessed if the severe colitis in BTK-KO mice was triggered by shift in their microbiota. As mucosal IgA-secretion is known to affect gut microbiota commensalism, we analyzed whether BTK-deficiency impaired gut mucosal IgA response by examining colon lamina propria (LP) IgA-expressing cells. We found the percentage and absolute numbers of B220^−^IgA^+^ cells in the colon LP to be comparable between WT and BTK-KO mice (Fig. 2A, B).

Since IgA-expressing cells are not perturbed in BTK-KO mice, we next assessed if BTK-KO mice harbored a microbiota that is different from WT mice through analyzing fecal bacteria composition via 16 S rRNA gene sequencing. Principal Coordinates Analysis (PCoA) showed clear separation of fecal bacterial communities between WT and BTK-KO mice (Fig. 2C), suggesting that they harbored distinct gut bacteria. To address if the exacerbated colitis in BTK-KO mice was due to this factor, we co-housed WT and BTK-KO mice to normalize their gut microbiota. After 4-week of co-housing at 1:1 ratio, we examined their fecal bacteria composition alongside singly-housed mice. We found that while the fecal bacteria communities from singly-housed WT and BTK-KO mice were different, fecal bacteria communities from co-housed WT and BTK-KO mice clustered together (Fig. 2D), indicating that cohousing improves gut microbiota similarity between them. Consistently, at individual species level, we identified that differentially abundant bacteria (*Bacteroides uniformis, Alistipes indistinctus and Rikenella microfusus*) showed obvious normalization upon cohousing (Fig. 2E). We next induced acute colitis in co-housed WT and BTK-KO mice by DSS-treatment and determined their disease susceptibility. Surprisingly, we found that after cohousing, BTK-deficient mice were still more susceptible to DSS-induced colitis, as indicated by the substantially higher rate of weight-loss and mortality (Fig. 2F, G). These results suggest that the more severe colitis seen in BTK-deficient mice is not due to their altered gut microbiota.

### Th1-driven inflammation is selectively enhanced in the gut of BTK-deficient mice

**Fig. 3:**
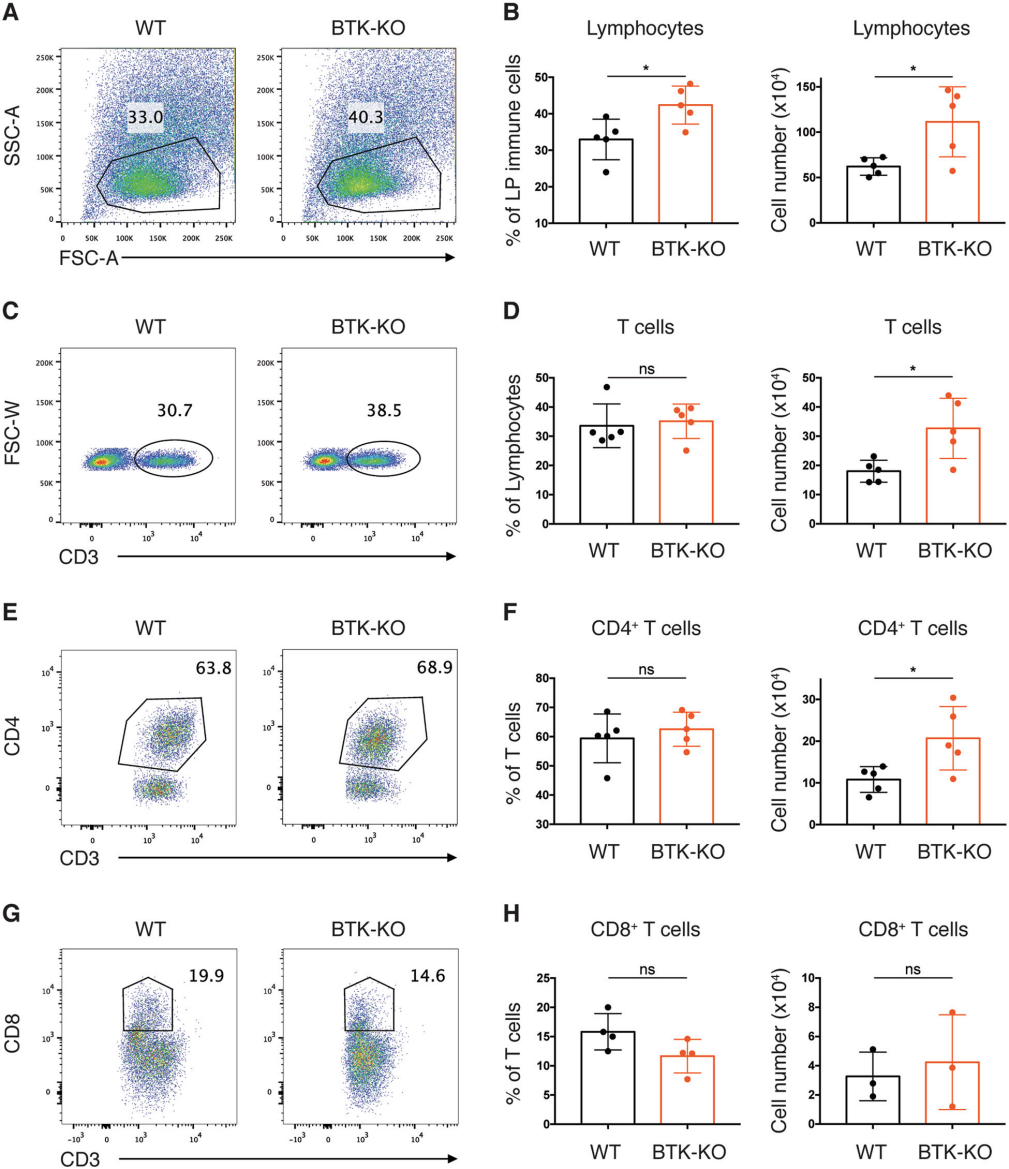
Increase in CD4^+^ T cell population in the gut LP of BTK-KO mice. **A** Colon LP immune cells from WT and BTK-KO mice gated for lymphocytes. Numbers shown are percentage of cells in the indicated gates. **B** Frequency (left) and total number (right) of lymphocytes in the immune cells from the colon LP of WT and BTK-KO mice. **C** Colon LP lymphocytes from WT and BTK-KO mice stained as indicated to reveal T cells. Numbers shown are percentage of cells in the indicated gates. **D** Frequency (left) and total number (right) of T cells in lymphocytes from colon LP of WT and BTK-KO mice. **E** Colon LP T cells from WT and BTK-KO mice stained as indicated to reveal CD4^+^ T cells. Numbers shown are percentage of cells in the indicated gates. **F** Frequency (left) and total number (right) of CD4^+^ T cells in total T cells from colon LP of WT and BTK-KO mice. **G** Colon LP T cells from WT and BTK-KO mice stained as indicated to reveal CD8^+^ T cells. Numbers shown are percentage of cells in the indicated gates. **H** Frequency (left) and total number (right) of CD8^+^ T cells in total T cells from colon LP of WT and BTK-KO mice. Data are presented as mean ± SD (*n* = 3–5). Statistical significance was determined by two-sided independent sample Student’s t test. ns: not significant; *P < 0.05, **P < 0.01 and ***P < 0.001.

We proceeded to examine whether BTK-deficiency affected the homeostasis of LP T cells, which are well-known regulators of gut immune homeostasis and inflammation. First, we analyzed colon LP lymphocytes from WT and BTK-KO mice by flow cytometry (Fig. 3A). Compared with WT mice, BTK-deficient mice had significantly higher percentage and total number of lymphocytes in the colon LP (Fig. 3B). As CD3^+^ T cells constitute ~30% of total lymphocytes in the colon LP of both WT and BTK-deficient mice, the number of CD3^+^ T cells also showed a significant increase in BTK-deficient mice (Fig. 3C, D). Furthermore, we found that in both WT and BTK-KO mice, the majority of T cells were CD4^+^ T cells, accounting for around 60% of total T cells; while CD8^+^ T cells only represented a minor fraction (<20%) of the total T cells (Fig. 3E–H). Interestingly, we found that the increased number of total T cells in BTK-deficient mice was mostly contributed by an increase in CD4^+^ T cells, which were doubled in the colon LP of BTK-KO compared to WT mice (Fig. 3F). However, the number of CD8^+^ T cells was comparable between WT and BTK-KO mice (Fig. 3H).

**Fig. 4:**
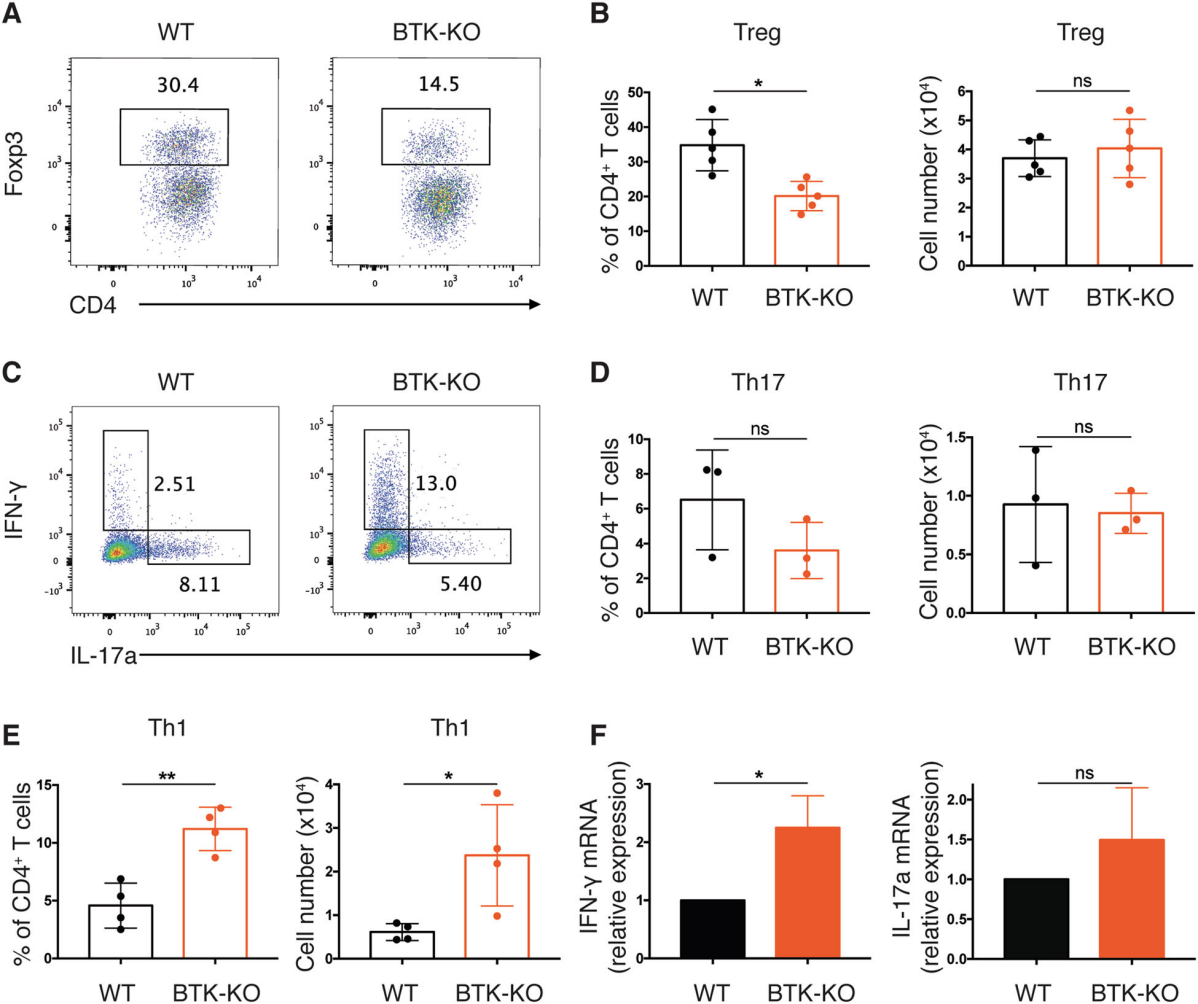
Th1 response is selectively enhanced in the gut LP of BTK-KO mice. **A** Colon LP CD4^+^ T cells from WT and BTK-KO mice stained as indicated to reveal Tregs (Foxp3^+^). Numbers shown are percentage of cells in the indicated gates. **B** Frequency (left) and total number (right) of Treg cells in CD4^+^ T cells from colon LP of WT and BTK-KO mice. **C** Colon LP CD4^+^ T cells from WT and BTK-KO mice stained as indicated to reveal Th1 (IFN-γ^+^) and Th17 (IL-17a^+^) cells. Numbers shown are percentage of cells in the indicated gates. **D** Frequency (left) and total number (right) of Th17 cells in CD4^+^ T cells from colon LP of WT and BTK-KO mice. **E** Frequency (left) and total number (right) of Th1 cells in CD4^+^ T cells from colon LP of WT and BTK-KO mice. **F** *IFN-γ* (left) and *IL-17a* (right) mRNA expression in colon LP immune cells from WT and BTK-KO mice analyzed by quantitative RT-PCR. GAPDH served as the internal control. Data are presented as mean ± SD (*n* = 3–5). Statistical significance was determined by two-sided independent sample Student’s *t*-test. ns: not significant; *P < 0.05, **P < 0.01 and ***P < 0.001.

As colon LP CD4^+^ T cells comprise both pro-inflammatory effector T-helper (Th, predominantly Th1 and Th17) and anti-inflammatory T-regulatory (Treg) cells, we determined which T cell subset(s) accounted for the increased CD4^+^ T cells in BTK-KO mice. First, we found that a major fraction (>30%) of CD4^+^ T cells were Foxp3^+^ Tregs in the WT mice, whereas it was reduced by half in BTK-KO mice (Fig. 4A, B). Interestingly, despite the reduced frequency, the number of Tregs were comparable between WT and BTK-KO mice (Fig. 4B). Similarly, there was a reduction (not statistically significant) in the frequency of IL-17a^+^ Th17 cells in BTK-KO mice, while the total number of Th17 cells were comparable (Fig. 4C, D). These data indicate that the reduced frequency of Tregs and Th17 cells in the colon LP of BTK-KO mice could be due to the expansion of other CD4^+^ T cell subsets. We next analyzed IFN-γ^+^ Th1 cells and found them to constitute a significantly higher percentage of the total CD4^+^ T cells in BTK-KO mice (Fig. 4C, E). Enumeration of cells revealed a 4-fold increase in the number of colon LP Th1 cells in BTK-KO mice compared with WT mice (Fig. 4E). Consistently, real-time quantitative PCR (RT-qPCR) analysis showed significantly increased *IFN-γ* but unaffected *IL-17a* expression in LP immune cells from BTK-KO mice (Fig. 4F). These data indicate that CD4^+^ T cell expansion in the colon LP of BTK-KO mice is due to increased Th1 cells and that Th1 response is selectively elevated in BTK-KO mice. Given that Th1 response is a major contributor to DSS-induced experimental colitis^18^, our results suggest that the increased colitis susceptibility in BTK-KO mice is attributed to their elevated gut Th1 response.

### Increased Th1 differentiation in lymphoid tissues of BTK-deficient mice

**Fig. 5:**
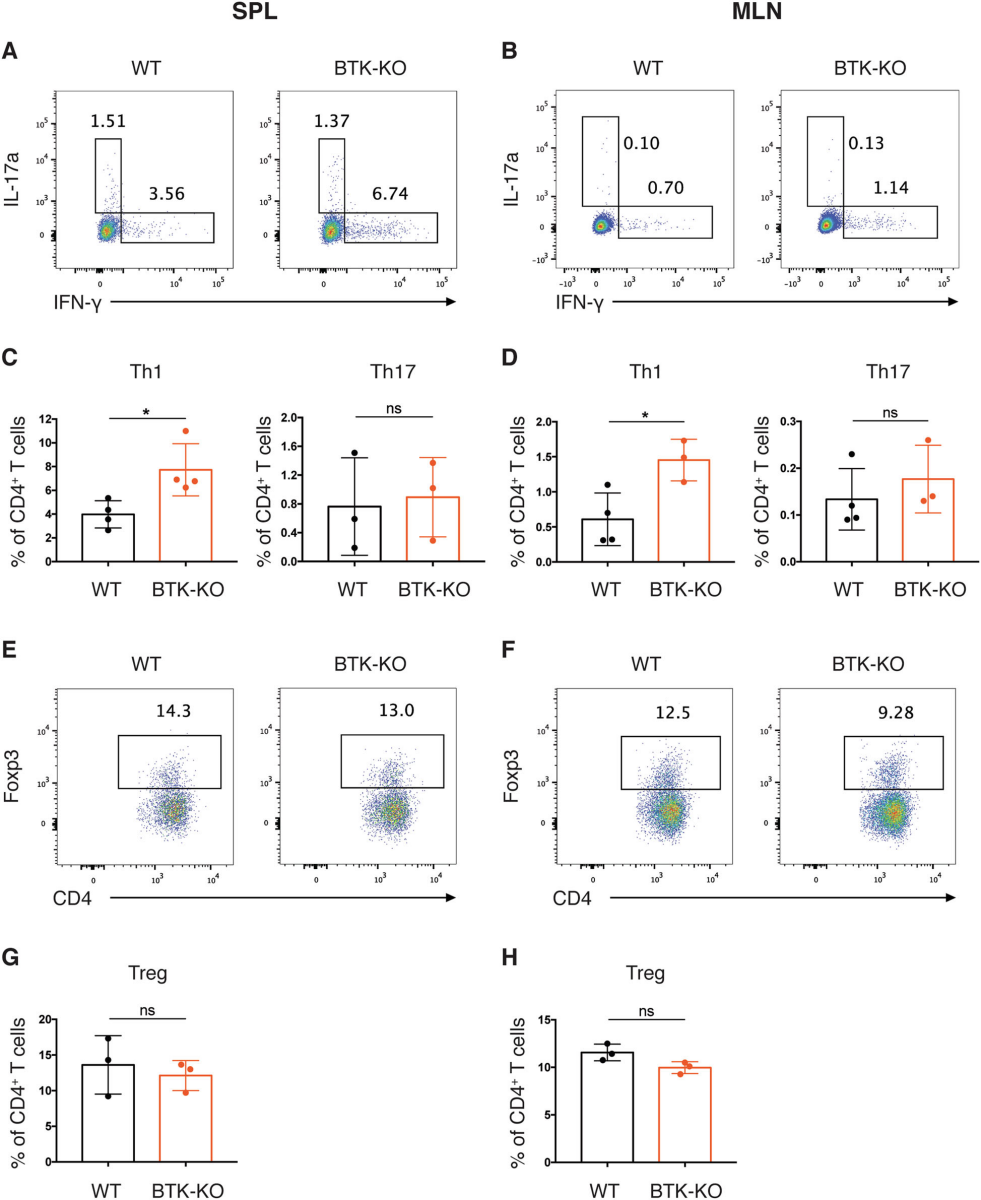
Increased differentiation of Th1 cells in lymphoid tissues of BTK-KO mice. CD4^+^ T cells from spleen (**A**) and MLNs (**B**) of WT and BTK-KO mice stained as indicated to reveal Th1 (IFN-γ^+^) and Th17 (IL-17a^+^) cells. Numbers shown are percentage of cells in the indicated gates. **C**, **D** Frequency of Th1 and Th17 cells in CD4^+^ T cells from the spleen (**C**) and MLNs (**D**) of WT and BTK-KO mice. **E**, **F** CD4^+^ T cells from spleen (**E**) and MLNs (**F**) of WT and BTK-KO mice stained as indicated to reveal Tregs (Foxp3^+^). Numbers shown are percentage of cells in the indicated gates. **G**, **H** Frequency of Treg cells in CD4^+^ T cells from the spleen (**G**) and MLNs (**H**) of WT and BTK-KO mice. SPL: spleen. MLN: mesenteric lymph node. Data are presented as mean ± SD (*n* = 3–4). Statistical significance was determined by two-sided independent sample Student’s t test. ns: not significant; *P < 0.05, **P < 0.01 and ***P < 0.001.

We next examined CD4^+^ T cells in other peripheral lymphoid tissues of BTK-KO mice. As most CD4^+^ T cells in the lymphoid tissues are naïve cells, we analyzed Th1 differentiation by assessing the frequency of Th1 cells in the total CD4^+^ T cell pool. Flow cytometric analysis revealed substantial increase in the frequency of IFN-γ^+^CD4^+^ T cells in the spleen and mesenteric lymph nodes (MLNs) of BTK-KO mice (Fig. 5A–D), indicative of increased Th1 cell differentiation in the spleen and gut-associated lymphoid tissues of BTK-KO mice. In contrast, we observed comparable Th17 and Treg populations in both the spleen and MLNs of WT and BTK-KO mice (Fig. 5A–H). Taken together, these data show that the differentiation of Th1 cells, but not Th17 or Tregs, is enhanced in the lymphoid tissues of BTK-KO mice and likely accounts for the increased Th1 response in their colon LP.

### Comparable innate immune cell populations in the gut of BTK-deficient mice

**Fig. 6:**
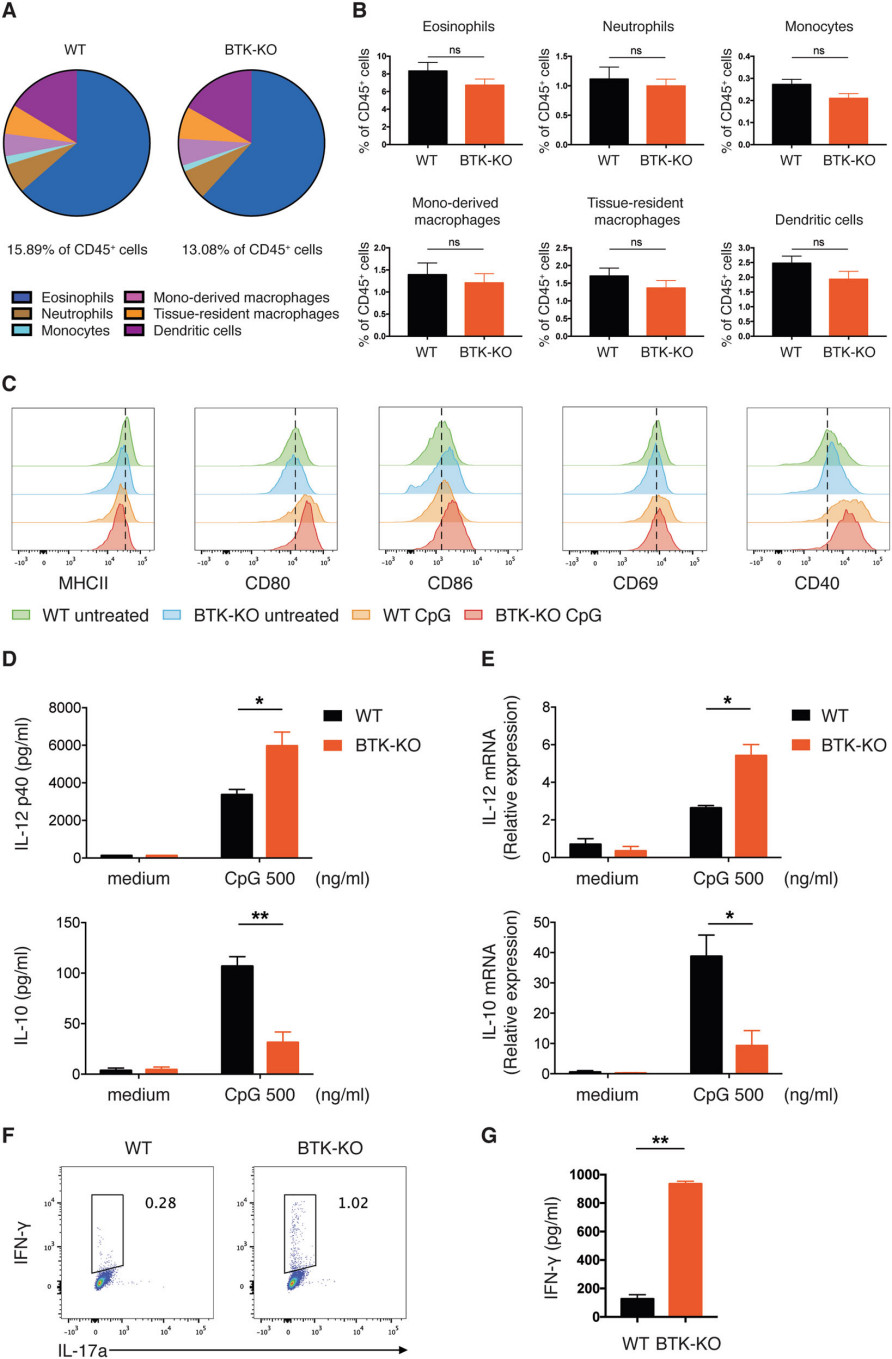
BTK-KO dendritic cells are more mature and produce higher amount of Th1 polarizing cytokine in response to TLR stimulation. **A** Composition of innate immune cell populations in colon LP of WT and BTK-KO mice. Mono-derived macrophage: monocyte-derived macrophage. **B** Frequency of individual innate immune cell populations in CD45^+^ cells from colon LP of WT and BTK-KO mice. **C**–**E** Maturation and cytokine production of splenic DCs from WT and BTK-KO mice. Splenic CD11c^+^ DCs from WT and BTK-KO mice were cultured in medium with or without 500 ng/ml CpG-ODN for 18 h. Expression of the indicated maturation markers on splenic DCs in response to CpG-ODN stimulation (**C**) was analyzed by flow cytometry. Data shown are representative of at least three independent experiments. IL-12p40 (upper) and IL-10 (lower) secretion from splenic DCs in response to CpG-ODN stimulation (**D**) was quantified by ELISA using known standards. *IL-12* (upper) and *IL-10* (lower) mRNA expression in splenic DCs in response to CpG-ODN stimulation (**E**) was analyzed by quantitative RT-PCR. GAPDH served as the internal control. **F**, **G** In vitro CD4^+^ T cell differentiation in DC-conditioned medium. Naïve CD4^+^ T cells were purified from spleen and MLNs of WT mice, followed by in vitro differentiation in medium prior conditioned by CpG-ODN-treated WT and BTK-KO DCs. After 5 days of differentiation, intracellular IFN-γ expression by differentiated CD4^+^ T cells (**F**) was analyzed by flow cytometry. IFN-γ secretion from differentiated CD4^+^ T cells into the culture supernatant (**G**) was quantified by ELISA using known standards. Data are presented as mean ± SEM [*n* = 5 for **D** upper panel, *n* = 4 for **D** lower panel, *n* = 3 for (**E**, **G**)]. Statistical significance was determined by two-sided independent sample Student’s t test. ns: not significant; *P < 0.05, **P < 0.01 and ***P < 0.001.

We next explored possible mechanisms by which BTK-deficiency leads to increased Th1 cell differentiation. We first investigated whether innate immune cell populations were perturbed in the gut of BTK-KO mice, given that these cells play crucial roles in T cell activation and differentiation. We examined innate immune cell populations in the colon LP following an established gating strategy (Supplementary Fig. 1a)^19^, and found that WT and BTK-KO mice had approximately equivalent percentage of total innate immune cells (collectively constituting 10~15% of CD45^+^ leukocytes) and a similar composition of innate cell subsets (Fig. 6A). Briefly, in mice of both genotypes, the most abundant colon LP innate cells were eosinophils (CD11b^+^F4/80^int^Ly6C^-^MHCII^−^), followed by dendritic cells (DCs) (CD11c^hi^MHCII^hi^), monocytes of different differentiation stages (i.e. CD11b^+^F4/80^int^Ly6C^hi^MHCII^−^ monocytes, CD11b^+^F4/80^int^Ly6C^+/^^−^MHCII^+^ monocyte-derived macrophages and CD11b^+^F4/80^hi^ tissue-resident macrophages) and neutrophils (CD11b^+^F4/80^low^). We found comparable frequencies and numbers of these innate immune cell populations in WT and BTK-KO mice (Fig. 6B, Supplementary Fig. 1b), indicating that the composition of innate immune cells is unaffected in the gut of BTK-KO mice.

### BTK-deficient dendritic cells promote Th1 differentiation in response to TLR stimulation

Next, we assessed if the function of innate immune cells is affected by BTK-deficiency. It has been documented that BTK is expressed in mouse DCs, which are the central antigen-presenting cells regulating T cell differentiation^20^. Due to limited availability of colon LP DCs and the observation that Th1 increase in the mutants was not exclusive to the gut, but also found in the spleen, we employed splenic CD11c^+^ DCs for the in-vitro functional analysis. TLR-stimulation is known to induce DC maturation and enhance their antigen-presenting capability, therefore we treated WT and BTK-KO splenic CD11c^+^ DCs with CpG-ODN, which stimulates TLR9. Flow cytometry analysis revealed that while unstimulated WT and BTK-KO splenic DCs expressed similar basal levels of MHCII, CD80 and CD69, BTK-KO DCs clearly displayed higher basal levels of CD86 and CD40 than WT DCs (Fig. 6C). As expected, CpG-ODN stimulation up-regulated these maturation markers on both WT and BTK-KO DCs, such that there was no difference in the expression levels of MHCII, CD80, CD69 and CD40 between WT and BTK-KO DCs. However, the expression level of CD86 was significantly higher in BTK-KO than WT DCs (Fig. 6C). These data suggest that BTK-KO DCs are phenotypically more mature than WT DCs.

TLR-stimulation also induces DCs to produce various cytokines critical for T cell differentiation. We examined the production of IL-12, an important cytokine driving Th1 polarisation^21^, and found BTK-KO DCs to secrete higher amount of IL-12 compared with WT DCs upon CpG-ODN stimulation (Fig. 6D). Consistently, our RT-qPCR analysis also showed that BTK-KO DCs upregulated *IL-12* expression to a greater extent compared with WT DCs after CpG-ODN stimulation (Fig. 6E). We also analyzed IL-10 secretion from WT and BTK-KO DCs upon CpG-ODN stimulation, as autocrine IL-10 production can modulate IL-12 expression in DCs^22^. We found BTK-KO DCs to produce less IL-10 upon CpG-ODN treatment (Fig. 6D, E), which could contribute to their elevated IL-12 secretion. Collectively, our results indicate that BTK negatively regulates TLR9-stimulated IL-12 production in DCs, possibly via promoting autocrine IL-10 production.

As BTK-KO DCs produced more IL-12 and less IL-10 in response to TLR9 stimulation, we next assessed whether this altered cytokine secretion affects Th1 cell differentiation. We cultured in vitro*-*activated naïve CD4^+^ T cells with conditioned medium harvested from CpG-ODN-stimulated WT or BTK-KO DCs. Flow cytometric analysis demonstrated that a higher percentage of CD4^+^ T cells was polarized to IFN-γ-expressing Th1 cells when they were cultured in BTK-KO DC-conditioned than in WT DC-conditioned medium (Fig. 6F). Our ELISA further revealed substantially higher amount of IFN-γ production from CD4^+^ T cells cultured in BTK-KO versus WT DC-conditioned medium (Fig. 6G). These data suggest that BTK-KO DCs produce a cytokine milieu more favorable for Th1 cell differentiation.

### BTK-deficient CD4^+^ T cells are more prone to Th1 differentiation

**Fig. 7:**
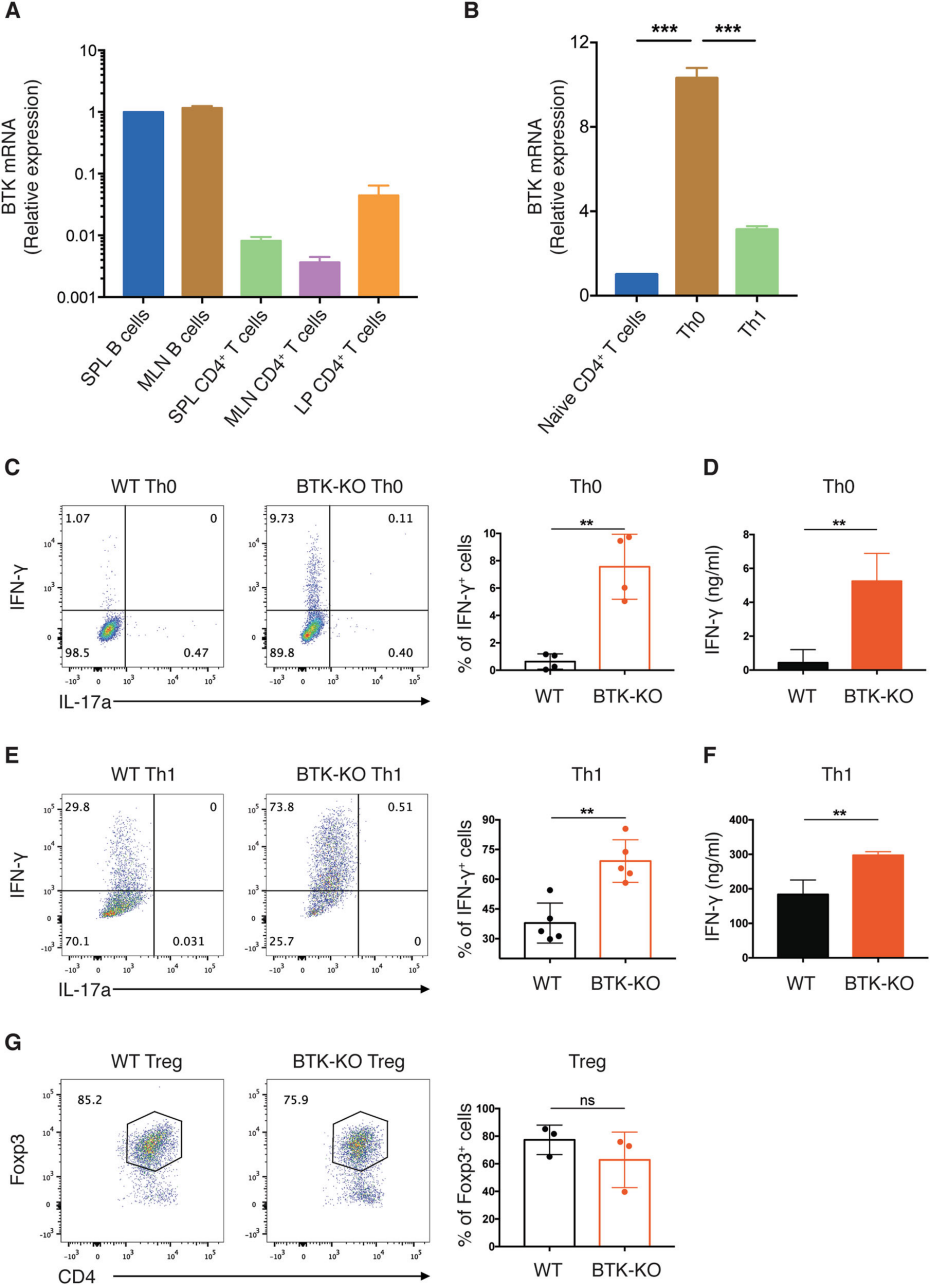
BTK-KO CD4^+^ T cells are more prone to Th1 differentiation. **A** *Btk* mRNA expression in the indicated cell subsets from WT mice analyzed by quantitative RT-PCR. SPL: spleen. MLN: mesenteric lymph node. LP: lamina propria. GAPDH served as the internal control. **B** *Btk* mRNA expression in naïve and in vitro differentiated CD4^+^ T cell subsets analyzed by quantitative RT-PCR. Ubiquitin C served as the internal control. **C**, **E** Intracellular IFN-γ expression by WT and BTK-KO CD4^+^ T cells differentiated under Th0 (**C**) and Th1 (**E**) conditions for 4 days. Numbers shown are percentage of cells in the indicated gates. **D**, **F** Quantification of IFN-γ secretion from CD4^+^ T cells differentiated under Th0 (**D**) and Th1 (**F**) conditions by ELISA using known standards. **G** Intracellular Foxp3 expression by WT and BTK-KO CD4^+^ T cells differentiated under the Treg condition for 3 days. Numbers shown are percentage of cells in the indicated gates. Data are presented as mean ± SD (*n* = 3–5). Statistical significance in **B** was determined by one-way ANOVA with Tukey’s multiple comparisons test. Statistical significance in **C**–**G** was determined by two-sided independent sample Student’s *t*-test. ns: not significant; *P < 0.05, **P < 0.01 and ***P < 0.001.

Recently, BTK was shown to be expressed in activated T cells^23^. Hence, we examined if BTK exerted a T cell-intrinsic role affecting Th1 differentiation. To confirm BTK expression in activated T cells, we sorted CD4^+^ T cells from spleen and MLNs, where naïve T cells are enriched, and from colon LP, where activated T cells reside. The expression of *Btk* in these T cells was determined by RT-qPCR. Compared with CD19^+^ B cells, *Btk* mRNA levels were significantly lower in CD4^+^ T cells from spleen and MLNs (Fig. 7A), consistent with previous studies showing undetectable *Btk* transcripts in T cells^11^. Interestingly, we found *Btk* mRNA level in colon LP CD4^+^ T cells to be 5- to 10-fold higher than in splenic and MLN CD4^+^ T cells, which was closed to ~5% of the *Btk* mRNA level expressed in B cell populations. Thus, these results suggest that there are low levels of BTK-expression in activated CD4^+^ T cells. Moreover, we demonstrated that *Btk* mRNA expression level was significantly increased in CD4^+^ T cells when they were activated in vitro under Th0 condition compared to the level in naïve CD4^+^ T cells (Fig. 7B). These results are consistent with recent publication, in which *Btk* mRNA level was shown to be 10-fold higher in effector/memory than in naïve CD4^+^ T cells^23^. Intriguingly, we found that when compared to Th0 condition, the induced *Btk*-expression was significantly suppressed in activated CD4^+^ T cells polarizing towards Th1 differentiation. These results imply that BTK-expression in activated CD4^+^ T cells could play an inhibitory role in Th1 differentiation.

Given reduced BTK-expression in CD4^+^ T cells polarizing into Th1 cells, we next asked if BTK-deficiency in CD4^+^ T cells could enhance their differentiation into Th1 cells. We purified naïve CD4^+^ T cells from lymphoid tissues of WT and BTK-KO mice and cultured them under Th0- and Th1-polarizing conditions. Interestingly, we found that under Th0 condition, a substantially higher percentage of BTK-deficient CD4^+^ T cells could differentiate into IFN-γ^+^ Th1 cells (Fig. 7C). ELISA analysis also detected increased amount of IFN-γ in the culture supernatant of BTK-KO CD4^+^ T cells under Th0 condition (Fig. 7D). When cultured under Th1 condition, with the addition of IL-12 and IL-2 in the culture medium, IFN-γ expression in CD4^+^ T cells was robustly induced (Fig. 7E). Again, we found that the percentage of IFN-γ-expressing cells was much higher in BTK-KO than WT CD4^+^ T cell cultures. Higher levels of IFN-γ were also detected in the culture supernatant of BTK-KO CD4^+^ T cells polarizing under Th1 condition (Fig. 7F). By contrast, we found that under Treg-polarizing condition, WT and BTK-KO CD4^+^ T cells were equivalent in differentiating into Foxp3^+^ Tregs (Fig. 7G), indicating that BTK-deficiency does not affect Treg cell differentiation. These data together suggest that BTK plays a T cell-intrinsic inhibitory role during Th1 cell differentiation and that BTK-deficiency promotes Th1 cell differentiation.

## Discussion

We show here that mice lacking BTK had increased susceptibility to DSS-induced colitis. Moreover, we found that normalization of BTK-deficient mice gut microbiota by cohousing them with WT mice did not reduce their enhanced susceptibility to DSS-induced colitis, thus ruling out altered microbiota as the main contributing factor for the more severe colitis in these mice. We further demonstrated that the gut LP of BTK-deficient mice have elevated Th1 cells, which are likely responsible for their enhanced colitis risk. Moreover, we found that the increase in Th1 response in BTK-deficient mice involves both T cell-extrinsic and -intrinsic mechanisms. We demonstrated that BTK-KO DCs were more mature and produced higher amounts of IL-12 upon TLR stimulation, which drives CD4^+^ T cell differentiation towards Th1 cells. Besides, we found BTK-expression to be induced in activated CD4^+^ T cells but suppressed in Th1 cells, suggesting that BTK plays an inhibitory role in Th1 cell differentiation. Consistently, we showed that activated BTK-deficient CD4^+^ T cells manifested a greater propensity to differentiate into IFN-γ-expressing Th1 cells. Thus, BTK-deficiency in CD4^+^ T cells likely promotes their differentiation into Th1 cells.

Our finding that DSS-induced colitis was exacerbated in BTK-deficient mice is consistent with a recent report showing that BTK-deficiency predisposed mice to TNBS-induced colitis^24^. To explore whether BTK-deficient mice harbor a disturbed gut microbiota driving colitis development, we first analyzed whether BTK-deficiency affects gut mucosal IgA response, an important regulator of microbiota commensalism^25,26^. Although BTK Thr316Ala missense mutation was previously implicated to cause IgA deficiency^27^, the numbers of IgA-expressing cells in the gut LP of BTK-deficient mice are not altered. Future investigations are needed to determine whether gut IgA responses are indeed unperturbed in BTK-deficient mice via the analysis of IgA affinity maturation and/or pattern of IgA-coating of intestinal microbes^28^. Our cohousing experiments further showed that the increased colitis risk in BTK-deficient mice was not induced by a colitogenic microbiota, as a reshaped microbiota similar to that seen in the WT mice did not rescue the severe disease phenotype in BTK-deficient mice. Here, we do not completely rule out the possibility that gut microbiota variation contributes to increased colitis risk in BTK-deficient mice, as 16 S rRNA sequencing analysis only assesses the gut bacteria. It would be interesting to study whether alterations in other gut microbiota components (e.g. viruses or fungi) might contribute to increased colitis susceptibility in the mutants.

Intriguingly, we discovered a pro-inflammatory CD4^+^ T cell landscape in the gut of BTK-KO mice, characterized by selective upregulation of Th1 response. Also, increased Th1 response was found in other lymphoid tissues of the mutants. Previous studies reported that BTK-deficient mice were resistant to parasite infections^29,30^, possibly through mounting a higher Th1 response. Frequent development of Th1-related autoimmune diseases has also been reported for XLA patients^31^. Here, for the first time, we demonstrate unequivocally that Th1 response is selectively enhanced in the gut mucosa of BTK-deficient mice, thus predisposing them to colitis. Indeed, the involvement of Th1 in colitis development has been well-documented. For example, CD4^+^CD45RB^hi^ T lymphocytes from normal donors adoptively transferred into immunocompromised mice lacking B and T cells could lead to colitis development with gut Th1 cell accumulation^32^, which could be attenuated with antibodies blocking Th1-related cytokines^33,34^. An increase in Th1-type cytokines has been also observed in DSS-induced acute colitis in mice^18^.

We found both T cell-extrinsic and -intrinsic mechanisms to be involved in the enhanced Th1 response in BTK-KO mice. Previous literature reported that BTK-deficiency promoted LPS-induced maturation of BMDCs^20^. Our results showed that BTK-deficient splenic DCs expressed higher levels of surface maturation markers than WT DCs before and after TLR9-stimulation, which may reflect a stronger T cell-stimulating ability of these mutant DCs. Besides, we found that BTK-deficiency enhances IL-12 and negatively impacts IL-10 in splenic DCs upon TLR9-stimulation, creating an altered cytokine milieu facilitating Th1 differentiation. Therefore, our data suggest that the combined effect of increased maturation and IL-12 production of BTK-deficient DCs is responsible for driving enhanced Th1 differentiation in BTK-KO mice.

In addition to T cell-extrinsic mechanism, our results also suggest that BTK regulates Th1 differentiation in a T cell-intrinsic manner. BTK and its analog, IL-2-inducible T-cell kinase (ITK), are known to mediate signaling downstream of BCR and TCR, respectively. The two kinases share structural and molecular function similarities. BTK-expression was previously undetected or detected at very low levels in T cells, while ITK is expressed almost exclusively in T cells^11,35–37^. This dogma, however, is challenged by research showing that T cells upregulated BTK-expression and BTK was phosphorylated upon TCR activation, suggesting functional importance of BTK in TCR signaling^23^. Our data further suggest that BTK plays an important role in regulating the polarization of activated CD4^+^ T cells into Th1 cells. Treatment of T cells with ibrutinib, an inhibitor of both BTK and ITK, is known to skew T-helper response towards Th1, an effect previously attributed solely to the inhibition of ITK in T cells^38^. Our data suggest that BTK may also play a role in attenuating Th1 cell differentiation. Future studies are required to elucidate the mechanisms of how T cell differentiation is regulated by intrinsic expression of BTK. In summary, our current study sheds new light on how BTK regulates gut immune homeostasis via affecting mucosal T cell responses.

## Material and methods

### Mice

WT (C57BL/6) and *Btk*^*−*/*−*^ mice were obtained from the Jackson Laboratory and bred in our facility. *Btk*^−/−^ mice were backcrossed for more than 10 generations to C57BL/6 background. Cohousing of WT and *Btk*^*−*/*−*^ mice was performed at 1:1 ratio for 4 weeks. Female mice of 8 to 12 weeks old with or without prior cohousing were used in the experiments. All animal experiments were conducted according to protocols approved by the Institutional Animal Care and Use Committee (IACUC) based on guidelines from the National Advisory Committee on Laboratory Animal Research.

### Dextran sulfate sodium (DSS)-induced colitis

Acute colitis in experimental mice was induced by adding DSS to drinking water. DSS (mol. wt. ~40,000, Sigma) was prepared at a concentration of 2% (w/v) with sterile drinking water and administered to mice ad libitum for 7 days. The DSS-containing water was subsequently replaced with sterile drinking water on day 8. Mouse survival and body weight were monitored daily. In all the experiments of DSS-induced colitis, mice showing clinical symptoms of debilitating disease (with weight loss >30% of original weight) and became moribund were sacrificed, complying with IACUC regulations.

### Histology

Colons were removed from unchallenged or DSS-treated (day 9) mice and fixed in 10% neutral buffered formalin. Paraffin embedded sections of the colon tissues were subjected to H&E staining and examined using the Evos FL Auto 2 Imaging System (Invitrogen). The histology score was assessed in a blinded manner using an established scoring system^39^, taking into account the degree of inflammatory cell infiltration and damage to the intestinal architecture.

### Fecal DNA extraction and 16 S rRNA gene sequencing

Total DNA from mouse stool samples was purified using Fast DNA Stool Mini Kit (QIAamp), quantified and sent for 16 S rRNA gene sequencing at the V3-V4 region using Ion S5XL platform. Sequencing data were analyzed by NovogeneAIT.

### Isolation of immune cells from colon LP, MLNs and spleen

To isolate LP immune cells, colons were excised, washed to remove feces, and PPs and fat were removed. Colons were then opened longitudinally, cut into 0.5 cm pieces, and shaken in Hank’s balanced salt solution (HBSS, life technologies) containing 5% fetal bovine serum (FBS, Biowest), 5 mM EDTA (Sigma) and 1 mM Dithiothreitol (DTT) for 20 min at 37 °C twice. The tissues were washed with HBSS and cut into small pieces, followed by digestion with RPMI1640 (Thermo Fisher Scientific) containing 10% FBS, 0.5 mg/ml collagenase D (Roche), 0.75 mg/ml Dispase 2 (Roche) and 50 μg/ml DNase 1 (Roche) for 15 min at 37 °C with shaking. The digested tissues were passed through 70μm cell strainers (Fisher Scientific). Digestion was repeated for three times until tissues were completely dissociated. Cells isolated from tissue digestion were centrifuged, washed in FACS buffer (5% FBS in PBS), re-suspended in 5 ml of 40% Percoll (GE Healthcare) and overlaid on 2.5 ml of 80% Percoll in a 15 ml tube. Percoll gradient centrifugation was performed at 1000 g for 20 min at 20 °C without brakes. Immune cells were collected from the interface of the 40/80 Percoll gradient, washed and resuspended in FACS buffer. Cells were counted and used immediately for subsequent experiments.

To isolate lymphocytes, MLNs and spleen were excised and gently grounded between glass slides to release single cells. Cells were harvested and incubated in red blood cell lysis buffer (0.15 M NH4Cl, 10 mM NaHCO3, 1 mM EDTA) for 5 min on ice. Cell suspensions were then passed through a 70 μm filter before being used for subsequent experiments.

### Isolation and in vitro TLR stimulation of splenic CD11c^+^ dendritic cells

CD11c^+^ dendritic cells were isolated from mouse spleen using CD11c MicroBeads Ultrapure, mouse kit (Miltenyi Biotec) according to manufacturer’s instructions. Purity of CD11c^+^ cells was determined by FACS to be >85%. In vitro TLR stimulation of splenic CD11c^+^ dendritic cells was performed by incubating 1million/ml cells at 37 °C for 18 h in complete RPMI medium (RPMI1640 supplemented with 10% FBS, 1% penicillin and streptomycin, 1 mM sodium pyruvate, 1 × MEM non-essential amino acids and 1 × 2-mercaptoethanol) in the presence or absence of CpG-ODN 1826 (500 ng/ml, InvivoGen). Cells incubated overnight were collected for total RNA isolation and flow cytometric analysis of surface maturation marker expression. The supernatant was collected for ELISA.

### Cell sorting by flow cytometry

Single cell suspensions prepared from spleen, MLNs and colon LP of WT mice were stained for surface markers for B (CD19^+^) and Th (CD3^+^CD4^+^) cells with fluorescein-conjugated antibodies before sorting of individual cell populations by flow cytometry (FACSAria II, BD Biosciences). Dead cells were excluded by staining with DAPI. The purities of the individual cell populations were higher than 98%. Sorted cells were lysed by TRIzol for RNA extraction.

### Purification and in vitro differentiation of naïve CD4^+^ T cells

Naïve CD4^+^ T cells were purified from mouse spleen and MLNs using Naive CD4^+^ T Cell Isolation Kit (Miltenyi Biotec). Purified naïve CD4^+^ T cells were plated at a density of 2.5 × 10^5^ cells/well at 37 °C in 48-well plates pre-coated with anti-CD3e (Clone: 145-2C11, 1 μg/ml, BD Biosciences) and anti-CD28 (Clone: 37.51, 1 μg/ml, BD Biosciences) antibodies. Activated cells were polarized under Th0 (no supplement), Th1 [IL-12 (5 ng/ml, R&D Systems), IL-2 (10 ng/ml, R&D Systems) and anti-IL-4 antibody (5 μg/ml, BD Biosciences)], Treg [TGF-β (5 ng/ml, R&D Systems), IL-2 (10 ng/ml, R&D Systems), anti-IFN-γ (5 μg/ml, BD Biosciences), anti-IL-12 p40/p70 (5 μg/ml, BD Biosciences) and anti-IL-4 (5 μg/ml, BD Biosciences) antibodies] differentiation conditions in complete RPMI medium or polarized in splenic DC-conditioned medium (overnight culture supernatant of CpG-ODN-stimulated splenic DCs).

### Total RNA isolation, reverse transcription (RT) and quantitative real-time PCR

Total RNA of cells was isolated using TRIzol (Invitrogen) according to manufacturer’s instructions. The mRNA in samples was reverse transcribed to its complementary DNA (cDNA) with Oligo(dT)_18_ primer (Invitrogen). The relative expression of IFN-γ, IL-17a, IL-10, IL-12b and BTK in the cDNA samples was analyzed by quantitative real-time PCR, using SYBR Select Master Mix (Thermo Fisher Scientific). Target genes were amplified for 40-60 cycles (95 °C for 15 s, 60 °C for 30 s) in QuantStudio 6 Flex Real-Time PCR Systems (Thermo Fisher Scientific). A melting curve was performed at the end of the PCR cycles to confirm the specificity of amplification. GAPDH and ubiquitin C were used as internal control, and relative expression of the target genes was calculated using ∆∆Ct. method. The primers used were listed in Supplementary Table 1.

### ELISA

The secretion of IL-12 and IL-10 from in vitro stimulated splenic DCs and secretion of IFN-γ from in vitro differentiated CD4^+^ T cells were measured by ELISA MAX™ Standard Mouse IL-12 p40, IL-10 and IFN-γ Kits (Biolegend) according to the manufacturer’s instructions.

### Intracellular staining of cytokines and transcription factors

Intracellular expression of cytokines in CD4^+^ T cells was analyzed using the Cytofix/Cytoperm Kit (BD Biosciences) according to the manufacturer’s instructions. Briefly, immune cells isolated from colon LP were incubated with 100 ng/ml PMA, 500 ng/ml ionomycin, and Golgistop (BD Biosciences) in complete RPMI medium at 37 °C for 4 h. Surface staining was performed with a cocktail of fluorescein-labeled antibodies for 20 min on ice. Cells were subsequently fixed and permeabilized with the Cytofix/Cytoperm solution for 20 min on ice and intracellular cytokine staining was performed with fluorescein-labeled cytokine antibodies for 30 min at room temperature (RT). The intracellular expression of Foxp3 in CD4^+^ T cells was analyzed using the Foxp3/Transcription Factor Staining Buffer Set (eBioscience).

### Flow cytometry

PE-Cy™7-CD4 (Clone: RM4-5), APC-Cy™7-CD69 (Clone: H1.2F3), APC-Cy™7-CD45 (Clone: 30-F11), APC-Cy™7-CD19 (Clone: 6D5), PE-Cy™7-MHC II (Clone: M5/114.15.2), PE-Foxp3 (Clone: MF-14), FITC-Ly6C (Clone: HK1.4), BV421-CD11b (Clone: M1/70) were purchased from Biolegend. PE-F4/80 (Clone: BM8), APC-IFN-γ (Clone: XMG1.2), Biotin-CD11c (Clone: N418) were purchased from eBioscience. APC-Cy™7-CD4 (Clone: GK1.5), APC-CD40 (Clone: HM40-3), APC-CD25 (Clone PC61), PerCP-Cy™5.5-CD80 (Clone: 16-10A1), FITC-CD3e (Clone: 145-2C11), PE-IL-17a (Clone: TC11-18H10), Sav-BUV395 were purchased from BD Biosciences. Flow cytometry data were acquired on BD LSR II flow cytometer as previously described^40^ and analyzed with FlowJo™ v10.7.

### Statistical analysis

Data are presented as mean ± SD or mean ± SEM as specified in the legend of each figure, with the number of replicates indicated in the legend of each figure. Statistical analysis was performed with GraphPad Prism 7 (GraphPad Software). Two-sided independent sample Student’s t test was used to compare the means between two groups, and one-way ANOVA with Tukey’s multiple comparisons test was used to compare the means between more than two groups. Log-rank (Mantel–Cox) test was used to determine statistical significance of the survival rate difference. *P*-value < 0.05 was considered significant. *P < 0.05, **P < 0.01 and ***P < 0.001.

## Supplementary information

Supplementary Figure

## References

[1] Kaser A, Zeissig S, Blumberg RS (2010). Inflammatory bowel disease. Annu. Rev. Immunol..

[2] Van Limbergen J, Wilson DC, Satsangi J (2009). The genetics of Crohn’s disease. Annu. Rev. Genomics Hum. Genet.

[3] Cho JH (2008). The genetics and immunopathogenesis of inflammatory bowel disease. Nat. Rev. Immunol..

[4] Goldstein DB (2009). Common genetic variation and human traits. N. Engl. J. Med.

[5] Glocker E-O (2009). Inflammatory bowel disease and mutations affecting the interleukin-10 receptor. N. Engl. J. Med.

[6] Garrett WS (2007). Communicable ulcerative colitis induced by T-bet deficiency in the innate immune system. Cell.

[7] Yao, X. et al. Remodelling of the gut microbiota by hyperactive NLRP3 induces regulatory T cells to maintain homeostasis. *Nat. Commun.* **8**, 1896 (2017). 10.1038/s41467-017-01917-2 PMC5711854 29196621

[8] Barmettler S (2017). Gastrointestinal manifestations in X-linked agammaglobulinemia. J. Clin. Immunol..

[9] Hendriks RW, Yuvaraj S, Kil LP (2014). Targeting Bruton’s tyrosine kinase in B cell malignancies. Nat. Rev. Cancer.

[10] de Weers M (1993). The Bruton’s tyrosine kinase gene is expressed throughout B cell differentiation, from early precursor B cell stages preceding immunoglobulin gene rearrangement up to mature B cell stages. Eur. J. Immunol..

[11] Smith CI (1994). Expression of Bruton’s agammaglobulinemia tyrosine kinase gene, BTK, is selectively down-regulated in T lymphocytes and plasma cells. J. Immunol..

[12] Xu, S., Lee, K. G., Huo, J., Kurosaki, T. & Lam, K. P. Combined deficiencies in Bruton tyrosine kinase and phospholipase Cγ2 arrest B-cell development at a pre-BCR^+^ stage. *Blood* **109**, 3377–3384 (2007). 10.1182/blood-2006-07-036418 17164342

[13] de Gorter DJ (2007). Bruton’s tyrosine kinase and phospholipase Cgamma2 mediate chemokine-controlled B cell migration and homing. Immunity.

[14] Jefferies CA (2003). Bruton’s tyrosine kinase is a Toll/interleukin-1 receptor domain-binding protein that participates in nuclear factor kappaB activation by Toll-like receptor 4. J. Biol. Chem..

[15] Liu X (2011). Intracellular MHC class II molecules promote TLR-triggered innate immune responses by maintaining activation of the kinase Btk. Nat. Immunol..

[16] Strober, W., Fuss, I. J. & Blumberg, R. S. The immunology of mucosal models of inflammation. *Annu. Rev. Immunol.* **20**, 495 (2002). 10.1146/annurev.immunol.20.100301.064816 11861611

[17] Sartor RB (2008). Microbial influences in inflammatory bowel diseases. Gastroenterology.

[18] Egger B (2000). Characterisation of acute murine dextran sodium sulphate colitis: cytokine profile and dose dependency. Digestion.

[19] Soncin, I. et al. The tumour microenvironment creates a niche for the self-renewal of tumour-promoting macrophages in colon adenoma. *Nat. Commun.* **9**, 582 (2018). 10.1038/s41467-018-02834-8 PMC5805689 29422500

[20] Kawakami Y (2006). Regulation of dendritic cell maturation and function by Bruton’s tyrosine kinase via IL-10 and Stat3. Proc. Natl Acad. Sci. USA.

[21] Koch MA (2012). T-bet(+) Treg cells undergo abortive Th1 cell differentiation due to impaired expression of IL-12 receptor beta2. Immunity.

[22] Lee KG, Xu S, Wong ET, Tergaonkar V, Lam KP (2008). Bruton’s tyrosine kinase separately regulates NFkappaB p65RelA activation and cytokine interleukin (IL)-10/IL-12 production in TLR9-stimulated B Cells. J. Biol. Chem..

[23] Xia S, Liu X, Cao X, Xu S (2020). T-cell expression of Bruton’s tyrosine kinase promotes autoreactive T-cell activation and exacerbates aplastic anemia. Cell Mol. Immunol..

[24] Mao, L. et al. Bruton tyrosine kinase deficiency augments NLRP3 inflammasome activation and causes IL-1β-mediated colitis. *J. Clin. Investig.* **130**, 1793–1807 (2020). 10.1172/JCI128322 PMC7108929 31895698

[25] Friman V, Nowrouzian F, Adlerberth I, Wold AE (2002). Increased frequency of intestinal Escherichia coli carrying genes for S fimbriae and haemolysin in IgA-deficient individuals. Micro. Pathog..

[26] Wei M (2011). Mice carrying a knock-in mutation of Aicda resulting in a defect in somatic hypermutation have impaired gut homeostasis and compromised mucosal defense. Nat. Immunol..

[27] Mitsuiki N (2015). Mutations in Bruton’s tyrosine kinase impair IgA responses. Int. J. Hematol..

[28] Honda K, Littman DR (2016). The microbiota in adaptive immune homeostasis and disease. Nature.

[29] Minoprio P, Coutinho A, Spinella S, Hontebeyrie-Joskowicz M (1991). Xid immunodeficiency imparts increased parasite clearance and resistance to pathology in experimental Chagas’ disease. Int. Immunol..

[30] Minoprio P (1993). Xid-associated resistance to experimental Chagas’ disease is IFN-gamma dependent. J. Immunol..

[31] Rosen FS, Cooper MD, Wedgwood RJ (1995). The primary immunodeficiencies. N. Engl. J. Med..

[32] Powrie F, Leach MW, Mauze S, Caddle LB, Coffman RL (1993). Phenotypically distinct subsets of CD4+ T cells induce or protect from chronic intestinal inflammation in C. B-17 scid mice. Int. Immunol..

[33] Claesson MH (1999). Colitis-inducing potency of CD4+ T cells in immunodeficient, adoptive hosts depends on their state of activation, IL-12 responsiveness, and CD45RB surface phenotype. J. Immunol..

[34] Liu Z (2001). Role of interleukin-12 in the induction of mucosal inflammation and abrogation of regulatory T cell function in chronic experimental colitis. Eur. J. Immunol..

[35] Liao XC, Littman DR (1995). Altered T cell receptor signaling and disrupted T cell development in mice lacking Itk. Immunity.

[36] Berg LJ, Finkelstein LD, Lucas JA, Schwartzberg PL (2005). Tec family kinases in T lymphocyte development and function. Annu. Rev. Immunol..

[37] Andreotti AH, Joseph RE, Conley JM, Iwasa J, Berg LJ (2018). Multidomain control over TEC kinase activation state tunes the T cell response. Annu. Rev. Immunol..

[38] Dubovsky JA (2013). Ibrutinib is an irreversible molecular inhibitor of ITK driving a Th1-selective pressure in T lymphocytes. Blood.

[39] Erben U (2014). A guide to histomorphological evaluation of intestinal inflammation in mouse models. Int. J. Clin. Exp. Pathol..

[40] Li, Y. F., Xu, S., Ou, X. & Lam, K. P. Shp1 signalling is required to establish the long-lived bone marrow plasma cell pool. *Nat. Commun.* **5**, 4273 (2014). 10.1038/ncomms5273 PMC4083441 24978161

