# Supplementary material for: Bruton’s tyrosine kinase regulates gut immune homeostasis through attenuating Th1 response
Source: Cell Death Dis. 2021 Apr 30;12(5):431. doi: 10.1038/s41419-021-03702-y (PMC8087775; doi:10.1038/s41419-021-03702-y)
Supplement: Supplementary file 1 — Supplementary Figure [file 41419_2021_3702_MOESM1_ESM.pdf]

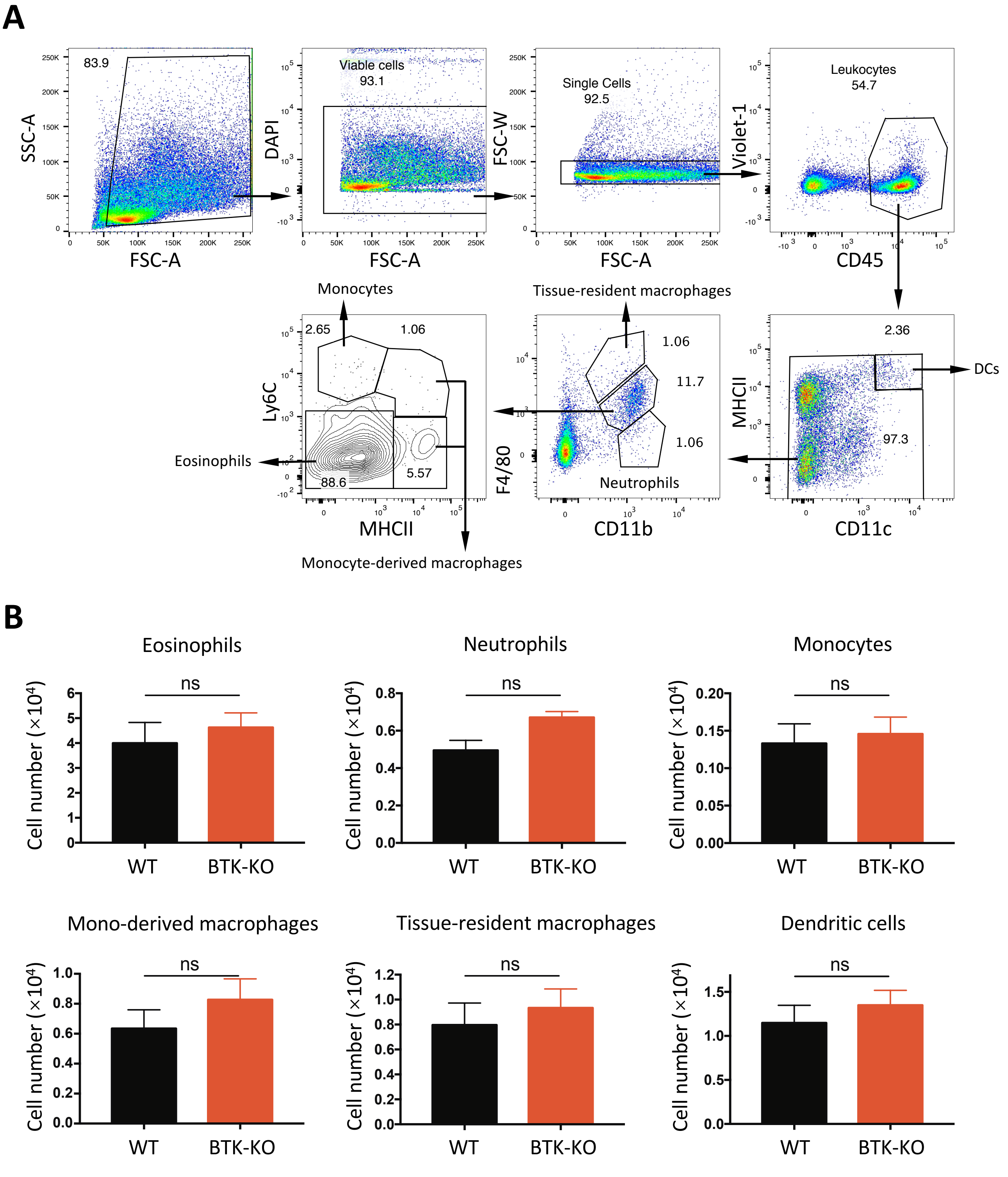

Supplementary Figure 1. Gating strategy and numbers of innate immune cell populations in the colon LP of WT and BTK-KO mice. (A) Gating strategy used for identifying innate immune cell populations in the mouse colon LP. Numbers shown are the percentage of cells in the indicated gates. **(B)** Numbers of innate immune cells in the colon LP of WT and BTK-KO mice. Data are presented as mean ± SEM (n = 4). Statistical significance was determined by two-sided independent sample Student's t test. ns: not significant.

Supplementary Table 1. Primers used for quantitative real-time PCR.

Target gene	Primers
Ifn-γ	F 5’-GGAACTGGCAAAAGGATGGTGA-3’
	R 5’-TGTTGCTGATGGCCTGATTGT-3’
Il-17a	F 5’-CCTGGACTCTCCACCGCAA-3’
	R 5’-AGCTTTCCCTCCGCATTGAC-3’
Btk	F 5’-GAGACCTGGCAGCTCGAAAC-3’
	R 5’-CGGACTGGAAACTTGGAGCC-3’
Il-10	F 5’-GGCCCAGAAATCAAGGAGCA-3’
	R 5’-AGAAATCGATGACAGCGCCTC-3’
Il-12b	F 5’-GCAGCAAAGCAAGATGTGTCC-3’
	R 5’-CTGTTTCTCCAGGGGCATCG-3’
